# Autoimmune pulmonary alveolar proteinosis co-existing with breast cancer: a case report

**DOI:** 10.1186/1752-1947-8-279

**Published:** 2014-08-19

**Authors:** Toyomitsu Sawai, Yasuhiro Umeyama, Sumako Yoshioka, Nobuko Matsuo, Naofumi Suyama, Shigeru Kohno

**Affiliations:** 1 Department of Respiratory Medicine, Nagasaki Harbor Medical Center City Hospital, 6-39 Shinchi-machi, Nagasaki 850-8555, Japan; 2 Second Department of Internal Medicine, Nagasaki University School of Medicine, 1-7-1 Sakamoto-machi, Nagasaki, Japan

**Keywords:** Breast cancer, Co-existing, Pulmonary alveolar proteinosis

## Abstract

**Introduction:**

Pulmonary alveolar proteinosis is a rare pulmonary disease characterized by excessive alveolar accumulation of surfactant due to defective alveolar clearance by macrophages. There are only a few published case reports of pulmonary alveolar proteinosis occurring in association with solid cancers. To the best of our knowledge, there are no previously reported cases of pulmonary alveolar proteinosis associated with breast cancer.

**Case presentation:**

A 48-year-old Asian woman, a nonsmoker, presented to our institution with a right breast mass. Biopsy examination of the lesion revealed scirrhous carcinoma. A chest computed tomography scan for metastases showed abnormal shadows in both upper lung fields. As a result of flexible fiberscopic bronchoscopy, this patient was diagnosed as having pulmonary alveolar proteinosis. This case was categorized as autoimmune pulmonary alveolar proteinosis due to the positive anti-granulocyte-macrophage colony-stimulating factor antibody. Pulmonary alveolar proteinosis decreased gradually after mastectomy.

**Conclusions:**

The present case involved the coincident occurrence of autoimmune pulmonary alveolar proteinosis with breast cancer; breast cancer may be a factor during pulmonary alveolar proteinosis development.

## Introduction

Pulmonary alveolar proteinosis (PAP), first described in 1958 [1], is an unusual disease characterized by the accumulation of large amounts of surfactant lipids and proteins in the alveolar spaces. This material may be responsible for pulmonary symptoms and impairment of gas exchange leading to respiratory failure. It has been observed both as an isolated process and in conjunction with a variety of inflammatory and neoplastic diseases of the hematopoietic and immune systems. There are only a few published case reports of PAP occurring in association with solid cancers [2-8].

## Case presentation

**Figure 1:**
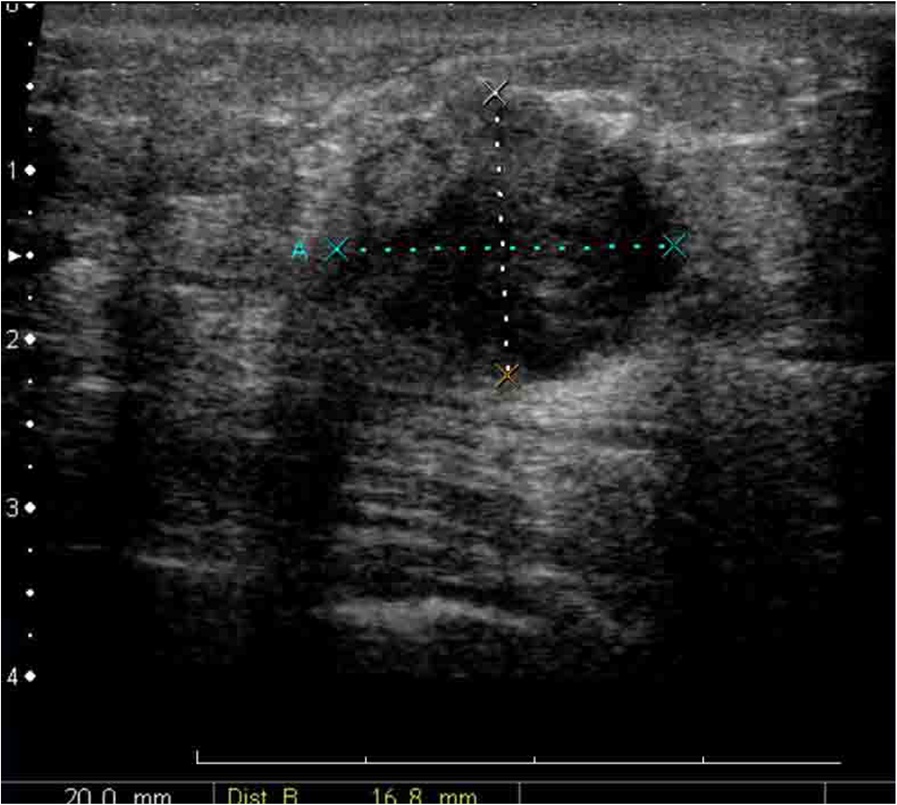
Ultrasonography shows an inhomogeneous, hypoechoic nodule measuring 20mm×17mm.

**Figure 2:**
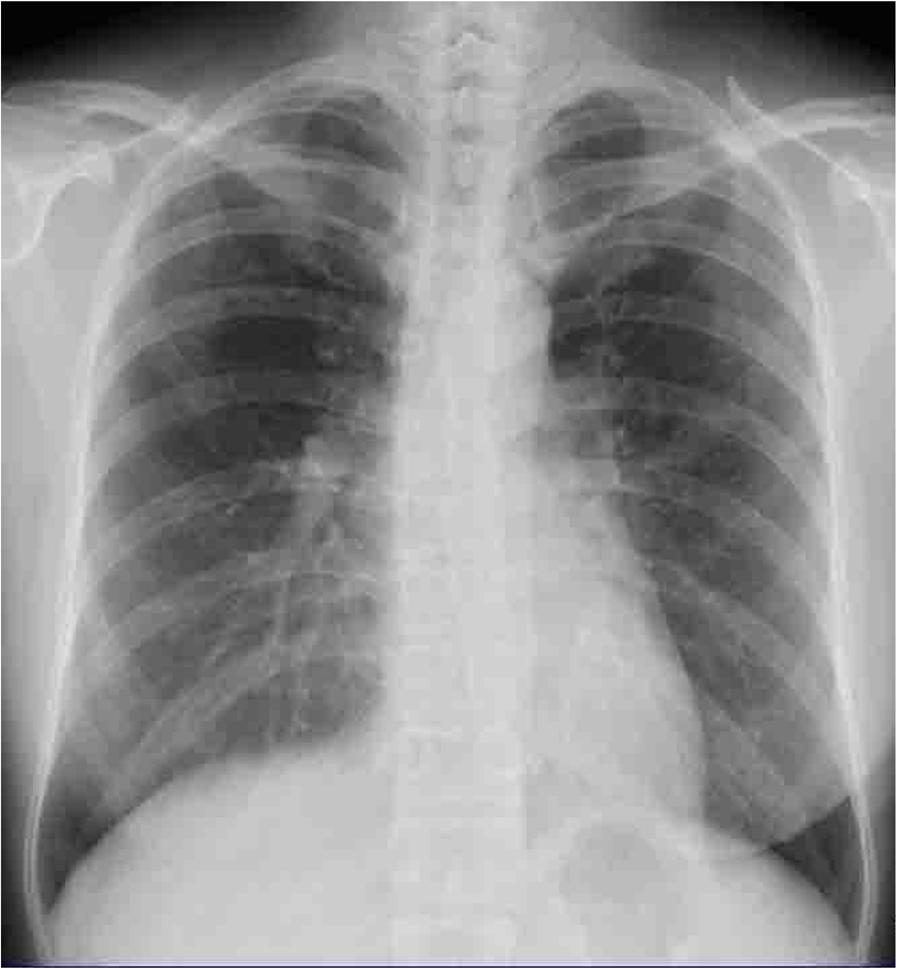
The chest X-ray shows slight peripheral infiltration shadows in both upper and middle lung fields.

**Figure 3:**
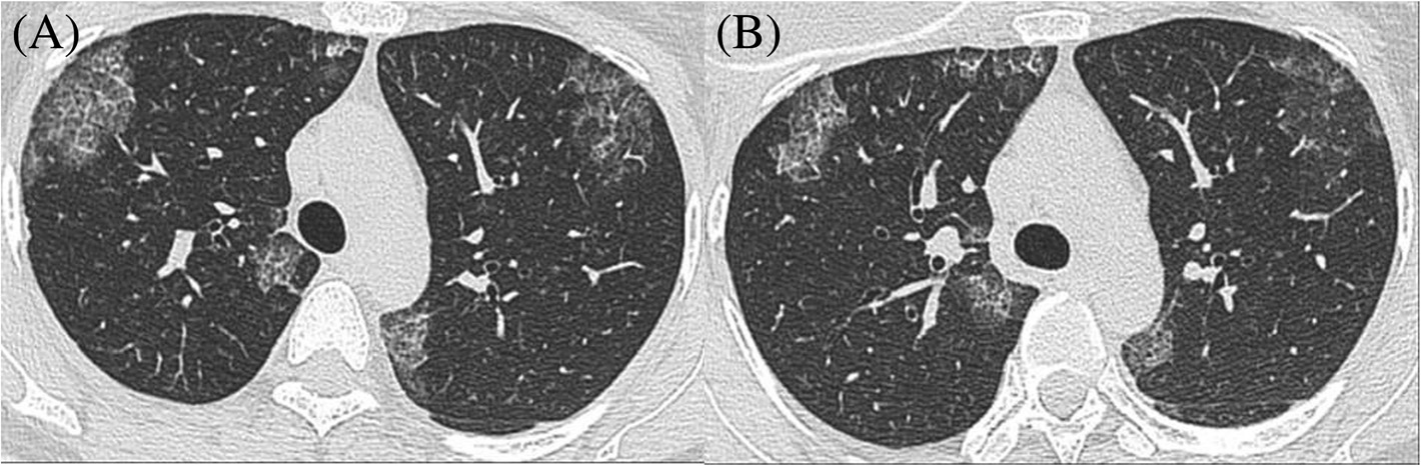
**Serial chest computed tomography shows gradual resolution. (A)** The chest computed tomography scan shows patchy peripheral ground-glass opacities and thickened interlobular septa in both upper lung fields (November 2013). **(B)** The chest computed tomography scan shows that the areas of ground-glass opacities and thickened interlobular septa representing pulmonary alveolar proteinosis have decreased (January 2014).

**Figure 4:**
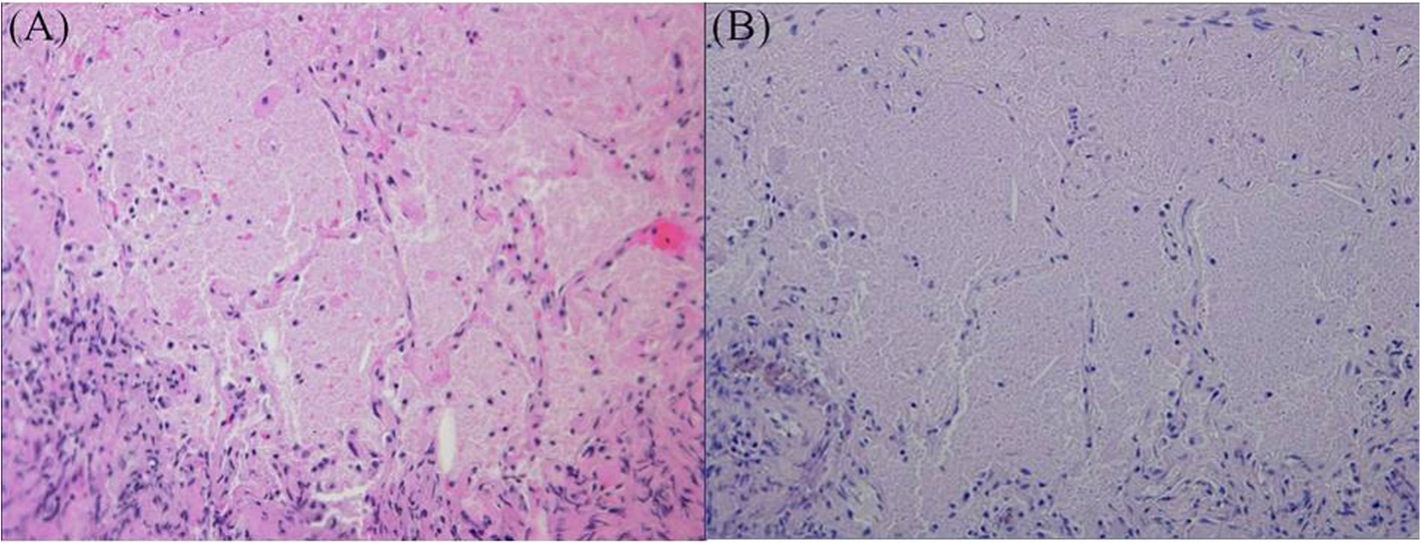
Histological findings of transbronchial lung biopsy specimens show the alveolar spaces to be filled with eosinophilic granular material (A; hematoxylin and eosin, ×200) and abundant intra-alveolar material that stains with periodic acid-Schiff (B; periodic acid-Schiff, ×200).

A 48-year-old, Asian woman, a nonsmoker, presented to our institution with a right breast mass. A physical examination revealed a hard mass in her right breast. There was no tenderness and no skin redness. The mass had a clear border and good mobility, and there was right axillary lymph node swelling. Ultrasonography displayed an inhomogeneous hypoechoic nodule measuring 20mm×17mm (Figure 1). Biopsy examination of the lesion revealed scirrhous carcinoma. A chest computed tomography (CT) scan for metastases showed abnormal shadows in both upper lung fields. The patient was then referred to our department for definitive workup and treatment. She had no history of cough, sputum, or dyspnea. Our patient had no history of tobacco smoking and no exposure to any dusts associated with a high risk of lung damage. Her past history and family history were unremarkable. A chest X-ray showed slight peripheral infiltration shadows in both upper and middle lung fields (Figure 2). A chest CT scan showed patchy peripheral ground-glass opacities and thickened interlobular septa in both upper lung fields (Figure 3A). A peripheral blood cell count and serum and biochemical tests were normal. Autoantibody and vasculitis screening was negative. Testing for human immunodeficiency virus infection was negative. Serum carcinoembryonic antigen (CEA) and carbohydrate antigen 15–3 (CA15-3) were in the normal ranges (1.1ng/mL and 12.5U/mL, respectively), but granulocyte-macrophage colony-stimulating factor (GM-CSF) autoantibody was elevated (29.57μg/mL). Pulmonary function testing revealed normal lung volumes and diffusing capacity. Flexible fiberscopic bronchoscopy was then performed. The retrieved bronchoalveolar lavage fluid (BALF) was transparent; it did not have a milky appearance. However, BALF cytology showed alveolar macrophages with granular materials that stained positively with periodic acid-Schiff (PAS). Histological findings of a transbronchial lung biopsy specimen showed the alveolar spaces to be filled with PAS-positive granular materials (Figure 4). As a result, this patient was diagnosed as having PAP.

Because she was asymptomatic and her oxygenation was good, a right modified radical mastectomy was performed. One month after surgery, the chest CT scan was repeated. It showed that the areas of ground-glass opacities and thickened interlobular septa representing PAP had decreased (Figure 3B). This patient required no further treatment after surgery. On follow-up, she had remained asymptomatic with respect to pulmonary disease, with no recurrence.

## Discussion

PAP is an unusual disease characterized by the accumulation of surfactant composed of proteins and lipids in pulmonary alveolar macrophages and alveoli due to defective alveolar clearance by macrophages. It has been observed both as an isolated process and in conjunction with a variety of inflammatory conditions such as infections, pneumoconiosis, and hematologic malignancies of the immune or hematopoietic systems, including leukemia, and lymphoma. In many of these conditions, defective or altered macrophage function has been demonstrated. Sakagami *et al.* reported that GM-CSF autoantibodies reproduce the pathologic manifestations of PAP in healthy macaques [9].

PAP is divided into the following three distinct clinical forms based on its etiology: autoimmune, secondary, and congenital [10]. Autoimmune PAP represents approximately 90 percent of PAP cases and is caused by neutralizing antibodies against GM-CSF. These populations are mostly normal hosts without underlying disease. Secondary PAP has been described in association with a variety of inflammatory and neoplastic diseases of the hematopoietic and immune systems that impair alveolar macrophage function, resulting in surfactant accumulation [11]. Congenital PAP is seen especially in children, and the radiological and clinical presentation depends on the gene mutations in encoding surfactant protein B or C or the ABCA3 transporter by the absence of GM-CSF receptor [12].

The association between secondary PAP and hematological disorders, mostly chronic myeloid leukemia, myelodysplastic syndrome, and lymphoma, is well established [11]. However, there have been only a few published case reports of PAP occurring in association with solid cancers, including five lung cancers, one metastatic pulmonary melanoma, one mesothelioma and one glioblastoma [2-8]. Of the eight cases, detection of GM-CSF autoantibodies was performed in only two lung cancer cases (Table 1); one was a case of autoimmune PAP with subsequent development of lung cancer [7], and the other was secondary PAP associated with lung cancer [8]. Liu *et al. *[8] suggested that the existence of some chemical immune inhibitors secreted from the lung cancer cells causing a local inhibitory effect on macrophages probably induced PAP. Furthermore, Athanassiadou *et al. *[13] reported that patients with primary lung cancer have a high number of functionally incompetent macrophages. In autoimmune PAP patients, Inoue *et al.* reported that four of 212 cases (1.9 percent) were associated with cancers, including lung cancer, colon cancer, prostatic cancer, and thyroid cancer [10]. Since the average age at diagnosis of PAP is 40 to 50 years, PAP with cancer may be rare. To the best of our knowledge, PAP with breast cancer has not been previously described. The present case of PAP co-existed with breast cancer, but this case was categorized as autoimmune PAP due to the positive anti-GM-CSF antibody. However, GM-CSF autoantibodies are also present in healthy persons and in immune globulin prepared from plasma obtained from healthy persons [9]. Certainly, high levels of GM-CSF autoantibodies are specifically associated with autoimmune PAP. Kitamura *et al.* reported that the mean level of the autoantibodies in the sera from 24 idiopathic (autoimmune) PAP patients was 180±22μg/mL, but the range was 35 to 430μg/mL [14]. The anti-GM-CSF antixbody of this patient was increased to 29.57μg/mL, but still less than 35μg/mL. Moreover, PAP decreased one month after breast cancer resection. A previous report found that significant spontaneous resolution of PAP occurred in 7.9 percent (24 of 303 cases) of patients [15], but the median time from diagnosis to resolution was 20 months. Thus, breast cancer may have been a factor during PAP development in this patient. Morgan *et al.* reported that breast cancer cells induced enhancement of osteoblast-stromal cells to increase prostaglandin E2 (PGE2) production, and the release of PGE2 downregulated GM-CSF production *in vitro *[16]. Liu *et al.* reported that overexpression of cytokeratin-associated protein (CAPC) in MDA-231 breast cancer cells downregulated nuclear factor κB (NF-κB) activity and its target genes, including GM-CSF, *in vitro *[17]. These findings suggest that the process of breast cancer causes a local inhibitory effect on macrophages.

**Table 1:** Clinical features of nine patients with solid organ cancer and pulmonary alveolar proteinosis reported in the literature

**Case/Ref**	**Sex/age (y)**	**Presentation**	**Clinical form**	**GM-CSF Ab**	**Cancer**	**Timing of cancer and PAP**
1/2)	M/24	Weight loss	Unknown	N.A.	Melanoma	Co-incident
2/3)	M/64	Lt. hemiplegia	Unknown	N.A.	Glioblastoma	Cancer first
3/4)	M/67	Cough, Sputum	Unknown	N.A.	Lung cancer (SCC)	Co-incident
4/5)	M/59	Cough, Dyspnea	Unknown	N.A.	Lung cancer (SCC)	Co-incident
5/6)	M/45	Chest pain	Unknown	N.A.	Mesothelioma	PAP first
6/7)	F/54	Dyspnea	Autoimmune	Positive	Lung cancer (Adeno)	PAP first
7/7)	F/59	Dyspnea	Unknown	N.A.	Lung cancer (Adeno)	Co-incident
8/8)	M/57	Cough, Sputum	Secondary	Negative	Lung cancer (SCC)	PAP first
9/our case	F/48	Asymptomatic	Autoimmune	Positive	Breast cancer	Co-incident

GM-CSF Ab, granulocyte-macrophage colony-stimulating factor antibody; PAP, pulmonary alveolar proteinosis; N.A., not available; SCC, squamous cell carcinoma; Adeno, adenocarcinoma.

## Conclusions

In conclusion, the first case of PAP co-existing with breast cancer was described. The present case involved the coincident occurrence of autoimmune PAP with breast cancer, but it is possible that breast cancer may be a factor during PAP development.

## Consent

Written informed consent was obtained from the patient for publication of this case report and any accompanying images. A copy of the written consent is available for review by the Editor-in-Chief of this journal.

## Abbreviations

ABCA3: ATP-binding cassette, sub-family A, member 3; BALF: bronchoalveolar lavage fluid; CA15-3: carbohydrate antigen 15–3; CAPC: cytokeratin-associated protein in cancer; CEA: carcinoembryonic antigen; CT: computed tomography; GM-CSF: granulocyte-macrophage colony-stimulating factor; NF-κB: nuclear factor κB; PAP: pulmonary alveolar proteinosis; PAS: periodic acid-Schiff; PGE2: prostaglandin E2.

## Competing interests

The authors declare that they have no competing interests.

## Authors’ contributions

TS managed the patient and reviewed the literature. YU contributed to the collection of patient data. SY and NM analyzed the radiologic findings. TS was the main writer of the manuscript. NS and SK moderated the manuscript. All authors read and approved the final manuscript.
